# P-906. Diagnosis, Treatment, and Outcomes in Patients with Central Nervous System Blastomycosis

**DOI:** 10.1093/ofid/ofae631.1097

**Published:** 2025-01-29

**Authors:** Megan Biggs

**Affiliations:** The Mayo Clinic, Rochester, Minnesota

## Abstract

**Background:**

*Blastomyces* is a dimorphic fungus found in the soil and is endemic to regions of the Ohio River basin and Southeastern United States. It can cause both local and systemic infections in several organs regardless of immunity status. Infection occurs through inhalation of aerosolized conidia. Blastomycoses most commonly affects the lungs and infection of the central nervous system (CNS) is rarely found representing approximately 5-10% of individuals with extra-pulmonary blastomycosis.

Demographics of Patients with CNS Blastomycosis

**Figure:**
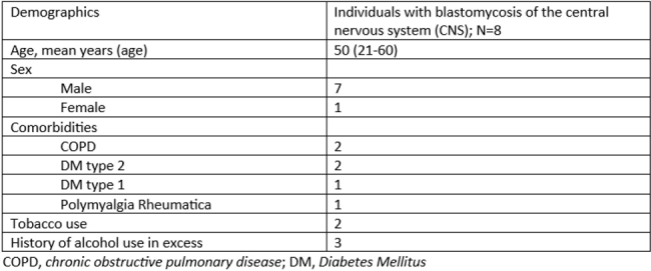

**Methods:**

Given the limited information on this rare form of blastomycoses, a retrospective review was conducted at a major tertiary center in Minnesota of adult inpatients diagnosed with proven or probable CNS blastomycoses between 2005 and 2020. Institutional Review Board approval at the Mayo Clinic was obtained. Data pertaining to diagnostic evaluation, treatment and outcomes were collected from eight medical charts found to meet criteria for proven or probable CNS Blastomycosis.

Diagnosis, Treatment, and Outcomes in Patients with CNS Blastomycosis

**Figure:**
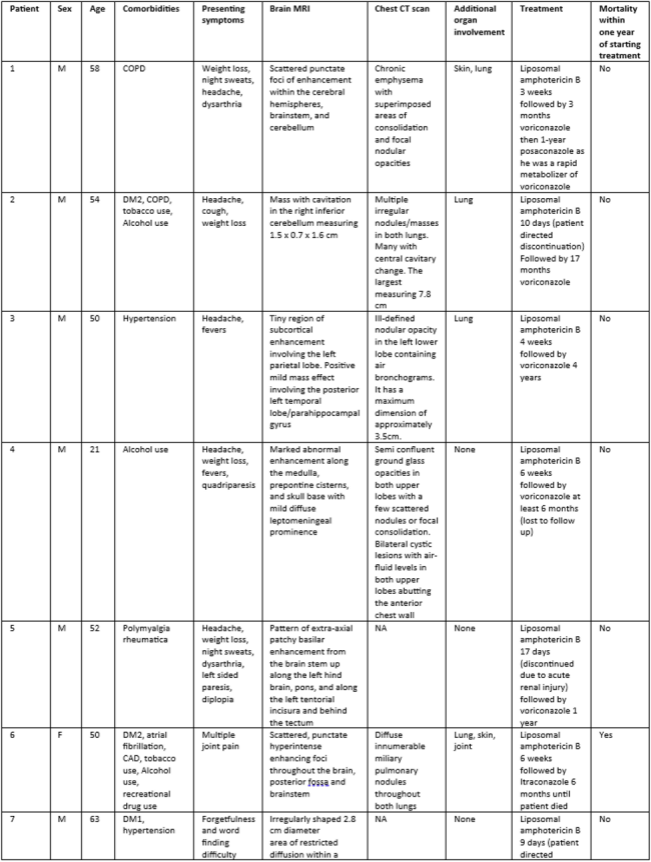

**Results:**

As seen in prior studies, most patients were male. The most common CNS related symptom on presentation was headache. Frequent comorbidities included diabetes, COPD, and alcohol use. Findings on MRI varied greatly. *Blastomyces* serum antibody was positive in only half of patients and urine antigen was frequently negative, however this test was not performed in one patient in this study. All but one patient survived to at least one year post diagnosis. All patients were treated with liposomal amphotericin B ranging from nine days to six weeks and voriconazole was the most frequently used azole. All patients treated with voriconazole survived at least one year following completion of treatment.

Diagnosis, Treatment, and Outcomes in Patients with CNS Blastomycosis (continued)

**Figure:**
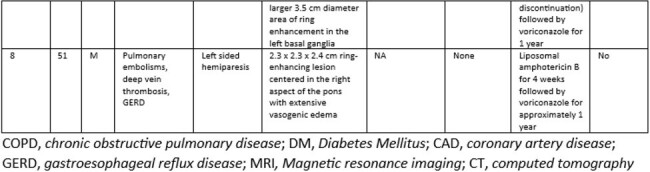

**Conclusion:**

This study emphasizes the importance of having a high suspicion when CNS symptoms are present in a patient with evidence of *Blastomyces*. Additionally, patients can have proven CNS blastomycoses without *Blastomyces* positive antibodies and/or antigen in blood or urine as well as variable MRI findings.

Laboratory and Culture Results in Patients Found to Have CNS Blastomyces

**Figure:**
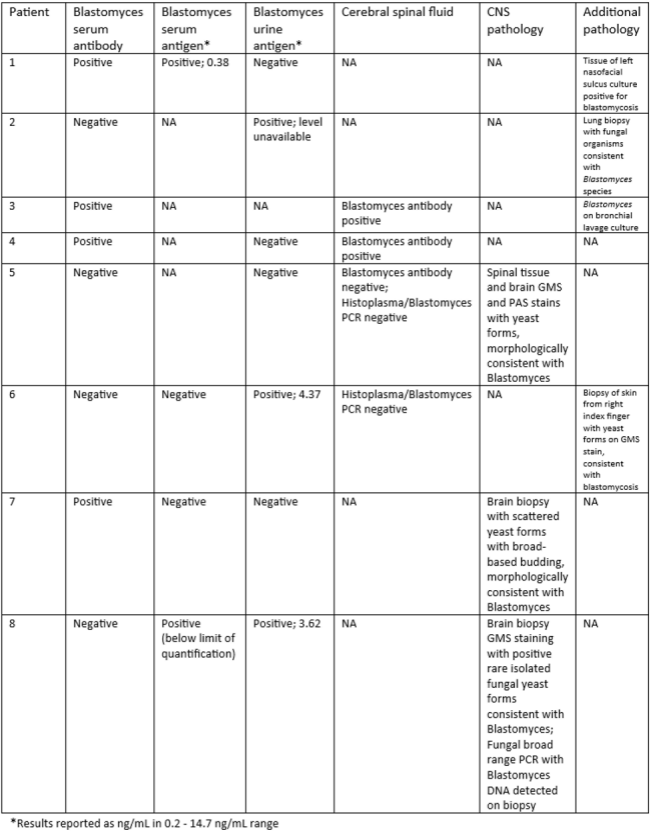

**Disclosures:**

**All Authors**: No reported disclosures

